# Paloma center of expertise - a national support system for refugees’ mental health

**DOI:** 10.1192/j.eurpsy.2021.1089

**Published:** 2021-08-13

**Authors:** M. Rautiokoski, J. Mäki-Opas, L. Somersalo, T. Halla

**Affiliations:** 1 Psychiatry, Tampere University Hospital, Tampere, Finland; 2 Equality And Inclusion Unit, Finnish Institute for Health and Welfare, Helsinki, Finland; 3 Mental Health Services, Tampere City, Tampere, Finland

**Keywords:** Refugees, Mental Health Services, Center of Expertise, Migration

## Abstract

**Introduction:**

Previous migrant population studies have shown that immigrants experience high level of psychological load and difficulties in accessing care. This is especially prevalent in those with refugee background. To tackle this issue, the PALOMA2 project (National support system for refugee mental health work and the knowhow dissemination) establishes a National PALOMA Centre of Expertise (PALOMA COE) for mental health work among refugees.

**Objectives:**

The PALOMA COE consists of all five University Hospital Areas and an NGO representative. The PALOMA COE work is becoming a permanent part of the Finnish health care structure. Each represented region has their own specific strengths and challenges, and the formation of Regional PALOMA COEs is planned accordingly. Together these Regional PALOMA COEs form the National PALOMA COE. Here we dive deeper into the Tampere University Hospital Region’s formation of PALOMA COE.

**Methods:**

Psychiatric Clinic for Refugees (PCR) has been working for over 24 years in the Tampere City area. PCR has a long history of PALOMA COE work in forms of clinical work, consulting and training professionals working with refugee mental health. From the beginning of 2021, PCR is integrating with Tampere University Hospital.

**Results:**

As a part of the integration process, the PALOMA COE work has a possibility to expand to the entire University Hospital area and better fulfill the specific needs of the entire region.

**Conclusions:**

The integration will improve the resources, quality and access to mental health care among people with refugee background.

